# Sketches from the Front

**Published:** 1919-10-11

**Authors:** 


### SKETCHES FROM THE FRONT.

One of the advantages of a citizen army is the variety of accomplishments of which any military unit is capable. Men of all trades are to be found in the ranks, and consequently work of a highly specialised nature can be done without extraneous help. Thus a gramaphone expert was discovered who mended the broken-down hospital gramaphones, and even a billiard-table mechanic was forthcoming to re-cover the little billiard-table in the patients' recreation hut. Among our own orderlies is a fair-haired, blue-eyed seaman; an invaluable man not merely because of his industry, but also because ours is a tent hospital, and he is constantly employed in repairing damaged tenting. A short while ago the writer observed him at the intake of a convoy of wounded, one cold and stormy night. He was acting as a stretcher-bearer and was dressed in oilskins. In reply to the comment that he was suitably clad he said, "Yes, it's a bad night, and there's plenty more bad weather to follow." "How do you know that?" "Well it's like this," he said. "At ten o'clock this morning there was a white ring round the sun, with sun 'elms, and that means we're going to have bad weather." "What is a sun 'elm?" "It's a little circle with faint colours like a rainbow, and you can see one to the north and one to the south of the ring round the sun. They always means bad weather. Besides, you may have noticed," he added, "that the wind was at first blowing from the norrard, then it became westerly, and later on it blew from the sou'-west, as though it were fighting against the sun. That's a bad sign, that is. Besides, a Friday's moon never was any good. She got up on a Friday and she'll go down on a Friday, and for the next fortnight we shall have bad weather." The writer is not weatherwise, and has never seen a sun 'elm, nor studied the ways of the moon in regard to the weather, but the few days that have passed since our friend from the sea made his sinister prognostications have satisfied fully his prophecy.

Among his other preliminary duties, a newly appointed general officer recently paid a visit to the various military hospitals in his command. The experience, apparently, was new to him, for he seemed a little shy and embarrassed in the wards of one of these institutions, and his jerky "How are you getting on?" with nothing further, at each bedside, had a staccato effect which began to make everyone present feel rather uncomfortable. At last the matron rose to the occasion as the procession halted opposite the bed of a pale and worn-looking wounded man, telling the general that the poor fellow had lost his right leg, while, curiously enough, his brother had been in the same ward a short while before, also having lost his right leg. "H'm," said the General as he moved away. "Anyhow, you've got a pair of legs between you." "No, sir," the man ejaculated. "How is that; what do you mean?" "They are both lefts, sir," was the prompt and comical reply.

